# UDP-*N*-Acetylglucosamine Pyrophosphorylase 2 (UAP2) and 1 (UAP1) Perform Synergetic Functions for Leaf Survival in Rice

**DOI:** 10.3389/fpls.2021.685102

**Published:** 2021-06-24

**Authors:** Zhaohai Wang, Qiang Wang, Lingxia Wei, Yan Shi, Ting Li, KeKe Hu, Shuai Liu, Hua Zhong, Jianglin Liao, Yangsheng Li, Hongyu Zhang, Yingjin Huang

**Affiliations:** ^1^Key Laboratory of Crop Physiology, Ecology and Genetic Breeding, Jiangxi Agricultural University, Ministry of Education of the People's Republic of China, Nanchang, China; ^2^Key Laboratory of Agriculture Responding to Climate Change, Jiangxi Agricultural University, Nanchang, China; ^3^Youth League Committee, Jiangxi Agricultural University, Nanchang, China; ^4^State Key Laboratory of Hybrid Rice, Key Laboratory for Research and Utilization of Heterosis in Indica Rice, Ministry of Agriculture, College of Life Sciences, Wuhan University, Wuhan, China; ^5^Department of Biochemistry, Molecular Biology, Entomology and Plant Pathology, Mississippi State University, Starkville, MS, United States

**Keywords:** UDP-N-acetylglucosamine pyrophosphorylase 2, UDP-N-acetylglucosamine pyrophosphorylase 1, defense response, early leaf senescence, rice (*Oryza sativa*)

## Abstract

Functional inactivation of UDP-*N*-acetylglucosamine pyrophosphorylase 1 (UAP1) induces defense response-related lesion-mimic spots and subsequent early senescence in every newly grown leaf of the rice mutant *uap1* after a short period's normal growth. However, the molecular mechanism of these leaves sustaining the short period's survival is still unknown. Phenotypic and molecular studies show that defense response-related lesion-mimic spots and early leaf senescence appear on the normally grown *uap1* leaf and aggravate with the growth time. Bioinformatic analysis reveals that UAP proteins are evolutionarily conserved among eukaryotes, and there exists UAP2 protein except UAP1 protein in many higher organisms, including rice. Rice UAP2 and UAP1 proteins present high sequence identities and very similar predicted 3D structures. Transcriptional expression profile of the *UAP2* gene decreases with the appearance and aggravating of leaf spots and early senescence of *uap1*, implying the role of the *UAP2* gene in maintaining the initial normal growth of *uap1* leaves. Enzymatic experiments verified that the UAP2 protein performs highly similar UAP enzymatic activity with the UAP1 protein, catalyzing the biosynthesis of UDP-GlcNAc. And these two UAP proteins are found to have the same subcellular localization in the cytoplasm, where they most presumably perform their functions. Overexpression of the *UAP2* gene in *uap1* plants succeeds to rescue their leaf mutant phenotype to normal, providing direct evidence for the similar function of the *UAP2* gene as the *UAP1* gene. The *UAP2* gene is mainly expressed in the young leaf stage for functions, while the *UAP1* gene is highly expressed during the whole leaf developmental stages. Based on these findings, it is suggested that *UAP2* and *UAP1* play key roles in rice leaf survival during its development in a synergetic manner, protecting the leaf from early senescence.

## Highlights

*UAP2* and *UAP1* coordinately expressed and colocalized into the cytoplasm to perform the UDP-*N*-acetylglucosamine pyrophosphorylase (UAP) enzymatic functions, maintaining rice leaf survival during its developmental process.

## Introduction

*N*-Acetylglucosamine (GlcNAc) is the fundamental amino sugar residue for the biosynthesis of *N*-glycan, which is essential for protein glycosylation (Stanley et al., 2015). *N*-Acetylglucosamine also acts as a sugar moiety in glycolipids (Raetz and Whitfield, 2002) and glycosylphosphatidylinositol (GPI)-anchor-linked protein (Hancock, 2004). UDP-GlcNAc is the active form of GlcNAc. The biosynthesis of UDP-GlcNAc and PPi from *N*-acetylglucosamine-1-phosphate (GlcNAc-1-P) and UTP is catalyzed by the enzyme *N*-acetylglucosamine-1-phosphate uridylyltransferase (GlcNAc1pUT) (Yang et al., 2010). And this enzyme is also named UDP-*N*-acetylglucosamine pyrophosphorylase (UAP) (Mio et al., 1998; Schimmelpfeng et al., 2006; Liu et al., 2013).

Mutants of the *UAP* gene have been found in various species. In *Escherichia coli* and *Mycobacterium tuberculosis, glmU* encodes the UAP protein, and the *glmU* mutants showed various alterations of cell shape and the final cell lysis (Mengin-Lecreulx and van Heijenoort, 1993; Zhang et al., 2008). In *Aspergillus fumigatus*, the conditional mutant of the *UAP1* gene showed defects in cell wall integrity and morphogenesis, and influenced the cell survival (Fang et al., 2013). In *Saccharomyces cerevisiae*, the null mutation of the *UAP1* gene was lethal, and most of the mutants showed fully swelled or lysed cells (Mio et al., 1998). In *Trypanosoma brucei*, the conditional null mutant of the *UAP* gene was unable to sustain growth under the non-permissive conditions (Stokes et al., 2008). In *Drosophila melanogaster*, the *UAP* gene mutants showed many phenotypic traits ranging from defects of the central nervous system fasciculation to defects in dorsal closure and eye development (Schimmelpfeng et al., 2006). In *Tribolium castaneum*, RNAi for *UAP1* resulted in a specific arrest at the larval–larval, larval–pupal, or pupal–adult molts, depending on the time of injection of double-stranded RNAs, whereas RNAi for *UAP2* prevented larval growth or resulted in pupal paralysis. And RNAi for either *UAP* gene at the mature adult stage resulted in the cessation of oviposition in females, as well as fat body depletion and eventual death in both sexes (Arakane et al., 2011). In *Locusta migratoria*, RNAi of *UAP1* resulted in 100% mortality, whereas insects with RNAi of *UAP2* were able to develop normally (Liu et al., 2013). In *Leptinotarsa decemlineata*, RNAi of *UAP1, UAP2*, and both genes made the larvae not undergo larvae–pupal ecdysis and be completely wrapped in the wrinkled larval cuticle, and finally die (Shi et al., 2016). And in *Arabidopsis thaliana*, the single mutants of *UAP1* and *UAP2* (also called *GlcNAc1pUT1* and *GlcNAc1pUT2*) revealed no obvious phenotype but their homozygous double mutant was lethal (Chen et al., 2014). It seems that the *UAP* gene plays an essential role in the cell or individual death in reported species.

Moreover, our previous study identified a *UAP1* gene mutant in rice, and a point mutation of the *UAP1* gene resulted in the complete functional inactivation of the UAP1 protein, leading to the appearance of defense response-related lesion-mimic spots and subsequent early leaf senescence for the *UAP1* gene mutant from the seedling stage (Wang et al., 2015). However, in these *uap1* mutant plants, every new leaf would grow normally for a period of time before these mutant phenotypes appear, thus making *uap1* plants sustain to the mature stage. And the molecular mechanism for the short period's survival of each new leaf on *uap1* plants still needs to be studied.

In this study, we report the identification and characterization of two rice *UAP* genes, *UAP2* and *UAP1*, about their synergetic functions in leaf survival at developmental stages. The UAP2 and UAP1 proteins have the same subcellular localization and highly similar enzymatic functions, while the gene expression profiling of the *UAP2* and *UAP1* genes determines the leaf destiny.

## Materials and Methods

### Plant Materials and Growth Conditions

The rice *UAP1* gene mutant *uap1*, also named *spl29* in our published paper (Wang et al., 2015), and its wild type, the rice cultivar “Zhonghua 11” (ZH11, *Oryza sativa* spp. *japonica*), were used in this study. After germination, rice seeds were grown in soil in the plant growth chamber (light cycle: 14-h light/10-h dark, 28°C) for seedling samples. For experiments at the tillering stage and on flag leaf development, rice plants were cultured under natural conditions.

### Gene Expression Analysis

Samples were collected, immediately frozen in liquid nitrogen, and then stored at −80°C for use. Total RNA of samples was extracted by using the TRIzol kit (Invitrogen, the United States), digested with the RNase-free DNase, and then used for preparing the cDNA templates with M-MLV reverse transcriptase (Promega, the United States). Using the SYBR Green Master Mix reagent (Bio-Rad, the United States), qRT-PCR was performed on a Bio-Rad CFX96 real-time PCR system, with three technological replicates for each biological sample. Four rice reference genes *UBC* (LOC_Os02g42314), *Profilin-2* (LOC_Os06g05880), *Actin1* (LOC_Os03g50885), and *ARF* (LOC_Os05g41060) were selected as internal standards for leaf samples (Wang et al., 2015, 2016). All primers used for qRT-PCR analysis are listed in Supplementary Table 1, with good PCR efficiencies (85–105%) assessed using a 10-fold dilution series of total cDNA.

### Alignment and Structure Comparison of UAP1 and UAP2 Protein Sequences

The *UAP1* and *UAP2* protein sequences were aligned with MAFFT-linsi v7.471 (Katoh and Standley, 2013). The Jalview (Waterhouse et al., 2009) was used to visualize the MSA. The standalone of I-TASSER software (Yang et al., 2015) was used to model the structure of *UAP1* and *UAP2* from rice. The top-fit models were selected based on the C-score provided by I-TASSER. Then, the best structures of two UAP proteins were visualized by PyMOL software. The protein structure comparison was made by TM-align (Zhang and Skolnick, 2005) online server (https://zhanglab.ccmb.med.umich.edu/TM-align/), and the TM-score value was used to scale the structural similarity with 1 indicating the excellent match.

### Recombinant Protein Construction, Expression, and Purification

To generate the glutathione S-transferase (GST) gene fusion constructs GST-UAP1 and GST-UAP2, the full-length coding sequence of *UAP1* and *UAP2* were amplified from the cDNA of ZH11 leaf, separately (primers GST-UAP1/UAP2 in Supplementary Table 2). PCR products were inserted into pGEX-6P-1 using the restriction enzyme sites *Bam*H I and *Eco*R I. Expression and purification of the recombinant protein were conducted according to the published method (Wang et al., 2015).

### ^1^H-Nuclear Magnetic Resonance Analysis of UAP1 and UAP2 Enzymatic Activities *in situ*

The enzymatic reactive experiments were performed according to the procedure as described previously (Wang et al., 2015). The forward reactions were carried out in the 540-μl mixture consisting of ^2^H_2_O/H_2_O (8:1, v/v), Na^+^/K^+^ phosphate buffer (80 mM K_2_HPO_4_, 20 mM NaH_2_PO_4_, pH 7.4), 5 mM MgCl_2_, 0.2 mM UTP, 0.2 mM GlcNAc-1-P, 1.5 units of yeast inorganic pyrophosphatase, and recombinant enzyme (0.5 μg of GST, GST-UAP1, or GST-UAP2). The reverse reactions were performed in a 540 μl solution containing ^2^H_2_O/H_2_O (8:1, v/v), Na^+^/K^+^ phosphate buffer, 5 mM MgCl_2_, 0.2 mM PPi, 0.2 mM UDP-GlcNAc (or UDP-GalNAc), and recombinant enzyme (0.5 μg of GST, GST-UAP1, or GST-UAP2). Examination of GlcNAc-1-P/GalNAc-1-P and UDPGlcNAc/UDP-GalNAc was performed by ^1^H-nuclear magnetic resonance (^1^H-NMR) as described previously (Zhang et al., 2011). Data acquisition started at 1 h after the addition of enzyme to the reaction mixture.

### Subcellular Localization of UAP1 and UAP2

The subcellular localization of the UAP1 and UAP2 proteins was predicted by using the online SignalP 4.1 Server (http://www.cbs.dtu.dk/services/SignalP-4.1/), ChloroP 1.1 Server (http://www.cbs.dtu.dk/services/ChloroP/), and TargetP 2.0 Server (http://www.cbs.dtu.dk/services/TargetP/).

To get the fusion construction of UAP1 and UAP2 with the yellow fluorescent protein (YFP), the coding sequences of UAP1 and UAP2 were amplified by using the primer pair UAP1-YFP and UAP2-YFP (Supplementary Table 2), and then cloned into the vector pBWD(LB)-p35SYFP using Bsa I restriction site. The fusion constructs (p35S::UAP1-YFP and p35S::UAP2-YFP) and the control (p35S::YFP) were transformed into the rice protoplasts for transient expression (Yu et al., 2014). And the subcellular localization results were examined using an FV1000 confocal system (OLYMPUS FLUOVIEW).

### Transgenic Plants

The *uap1* transgenic lines with the *UAP1* gene complementary vector (also named p*SPL29*C) were obtained from our previous research (Wang et al., 2015). For overexpressing the *UAP2* gene in *uap1*, the full-length coding sequence of the *UAP2* was amplified using primers “*UAP2*-OE” (Supplementary Table 2). PCR products were inserted into the binary vector pBWA(V)BU (reconstructed from pCAMBIA3300) using *Bsa* I sites for the digesting-link one-step reaction. The recombinant vectors were transferred into *Escherichia coli* DH5α and then sequenced to check whether the constructions were correct. The correct construction vector with *UAP2* and the empty vector were separately introduced into *Agrobacterium tumefaciens* EHA105 and then transformed into the *uap1* calli. Positive transgenic plants were confirmed by PCR amplifying the phosphinothricin gene with primer “*Bar178*” (Supplementary Table 2) and survival screening with the phosphinothricin solution (20 mg/L).

## Results

### Lesion-Mimic Spots and Early Leaf Senescence Appear on the Newly Developed Leaves of the *uap1* Mutant After a Short Period's Normal Growth

**Figure 1:**
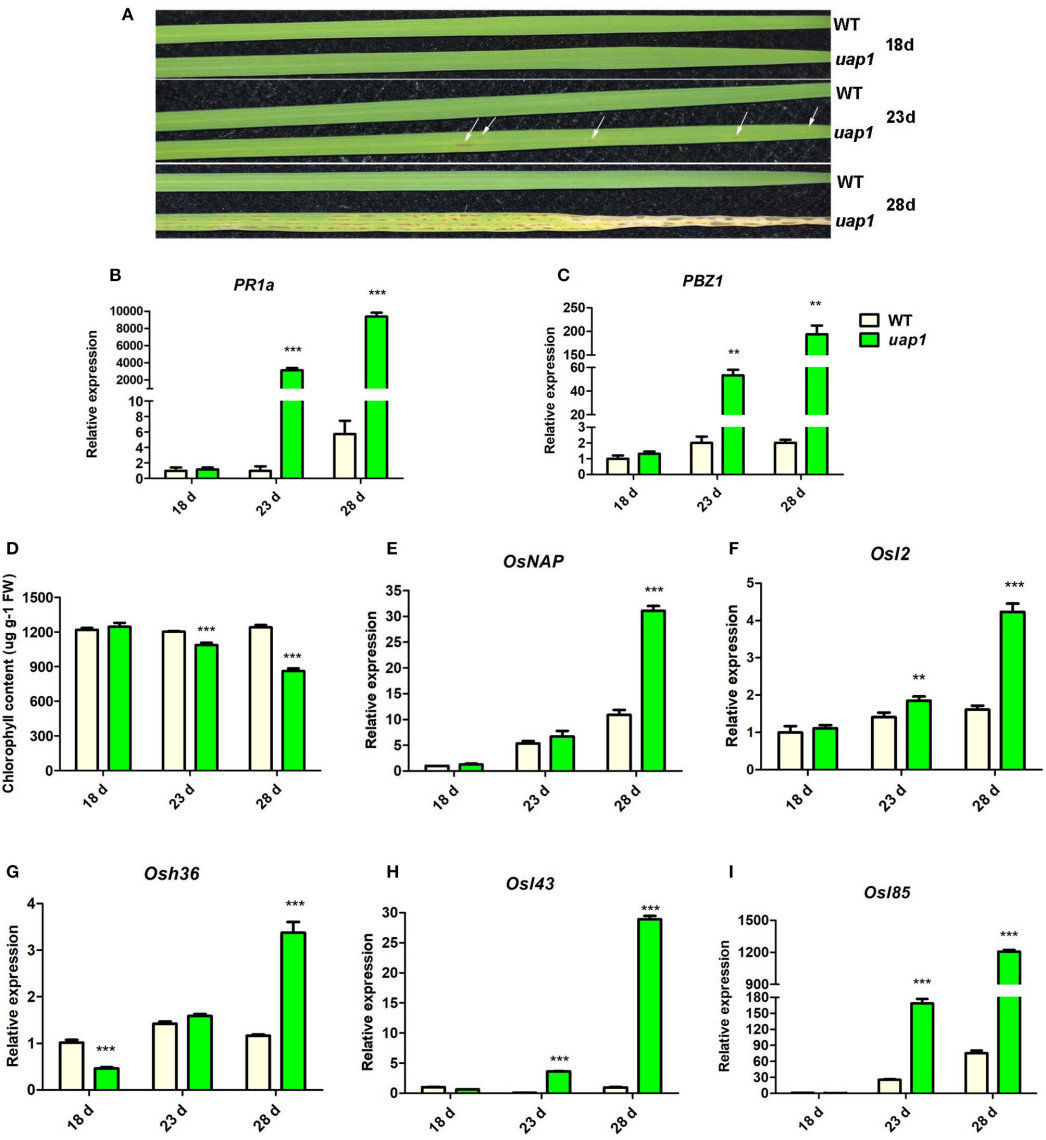
Characteristics of phenotype and molecular markers of wild-type and *uap1* mutant leaves. **(A)** Leaf phenotype of plants at the seedling stage (18, 23, and 28 days after germination). The white arrows indicate the leaf spots. **(B,C)** Relative expression of two defense signaling-related genes *PR1a* and *PBZ1*. **(D)** Chlorophyll contents. FW, fresh weight. **(E)** Relative expression of senescence-associated transcriptional factor *OsNAP*. **(F–I)** Relative expression of four senescence-associated genes (SAGs) *Osl2, Osh36, Osl43*, and *Osl85*. Relative expression of genes by qRT-PCR is normalized with reference genes *UBC, Profilin-2*, and *Actin1*. All data represent the mean ± *SD* of three biological replicates, and the asterisk indicates the statistically significant difference between *uap1* and WT (**P < 0.005, ***P < 0.0005, Student's *t*-test).

The *uap1* mutant appears to have the phenotype of lesion-mimic leaf spots and early leaf senescence after a period of normal growth. When the wild-type and *uap1* plants were grown in the plant growth chamber (14-h light/10-h dark, 28 °C), there was no visible mutant phenotype in leaves of the 18-day-old *uap1* plants (Figure 1A). However, small, dark-brown lesion-mimic leaf spots started to appear on the 23-day-old *uap1* plant leaves (Figure 1A). Soon, amounts of leaf spots spread over the 28-day-old *uap1* plant leaves; meanwhile, the leaves started to wither from the tip (Figure 1A).

The appearance of lesion-mimic leaf spots usually implies induced defense responses for plant resistance, and defense response genes will be activated in this process (Lorrain et al., 2003). In order to identify the defense response state of *uap1* leaves, gene expression analysis of two defense response genes (*PR1a* and *PBZ1*) was performed by qRT-PCR. Results showed that the expression levels of these two genes were equal in 18-day-old *uap1* and wild-type plant leaves, but gradually and significantly increased in leaves of 23- and 28-day-old *uap1* plants compared with the wild type (Figures 1B,C). These results showed that the defense response state in *uap1* leaves is originally normal, but is activated along with the appearance of leaf spots and aggravated with spots spreading.

Since early leaf senescence followed after the appearance of leaf spots in *uap1* mutant, this phenotype was additionally verified at the physiological and molecular level. The decline of chlorophyll content is an important physiological index of leaf senescence. Compared with the wild type, the chlorophyll content did not change in leaves of 18-day-old *uap1* plants, but it decreased in leaves of 23-day-old *uap1* plants, and continuously reduced in leaves of 28-day-old *uap1* plants (Figure 1D). Transcription factor genes and senescence-associated genes (SAGs) are usually up-regulated during leaf senescence to trigger or control the process (Lee et al., 2001; Liang et al., 2014). To further confirm that senescence occurred at the early stage of leaf developmental process of *uap1* plants, gene expression analysis of the senescence-associated transcription factor *OsNAP* and four SAGs (*Osl2, Osh36, Osl43*, and *Osl85*) was performed by qRT-PCR. Compared with the wild type, the mRNA levels of these genes were not raised in leaves of the 18-day-old *uap1* plants, but showed the upward trend in leaves of the 23-day-old *uap1* plants, and were all significantly up-regulated in leaves of the 28-day-old *uap1* plants (Figures 1E–I). The up-regulated expression patterns of senescence-associated transcription factor and SAGs further support the notion that early leaf senescence of *uap1* plants appeared from nothing during the process of young leaf development to mature.

### Analysis on Evolutionary Relationship and Expression Profile Implies That the *UAP2* Gene Is Responsible for the Short Time's Normal Growth of the *uap1* Young Leaves

Previous study has verified that the mutation of *UAP1* to *uap1* makes its encoded protein eliminate the UAP enzymatic function, responsible for the early leaf senescence, and defense response phenotype of the mutant (Wang et al., 2015). However, it is still not clear why the leaves of *uap1* mutant can grow normally for a period of time, despite the lost function of the UAP1 protein.

**Figure 2:**
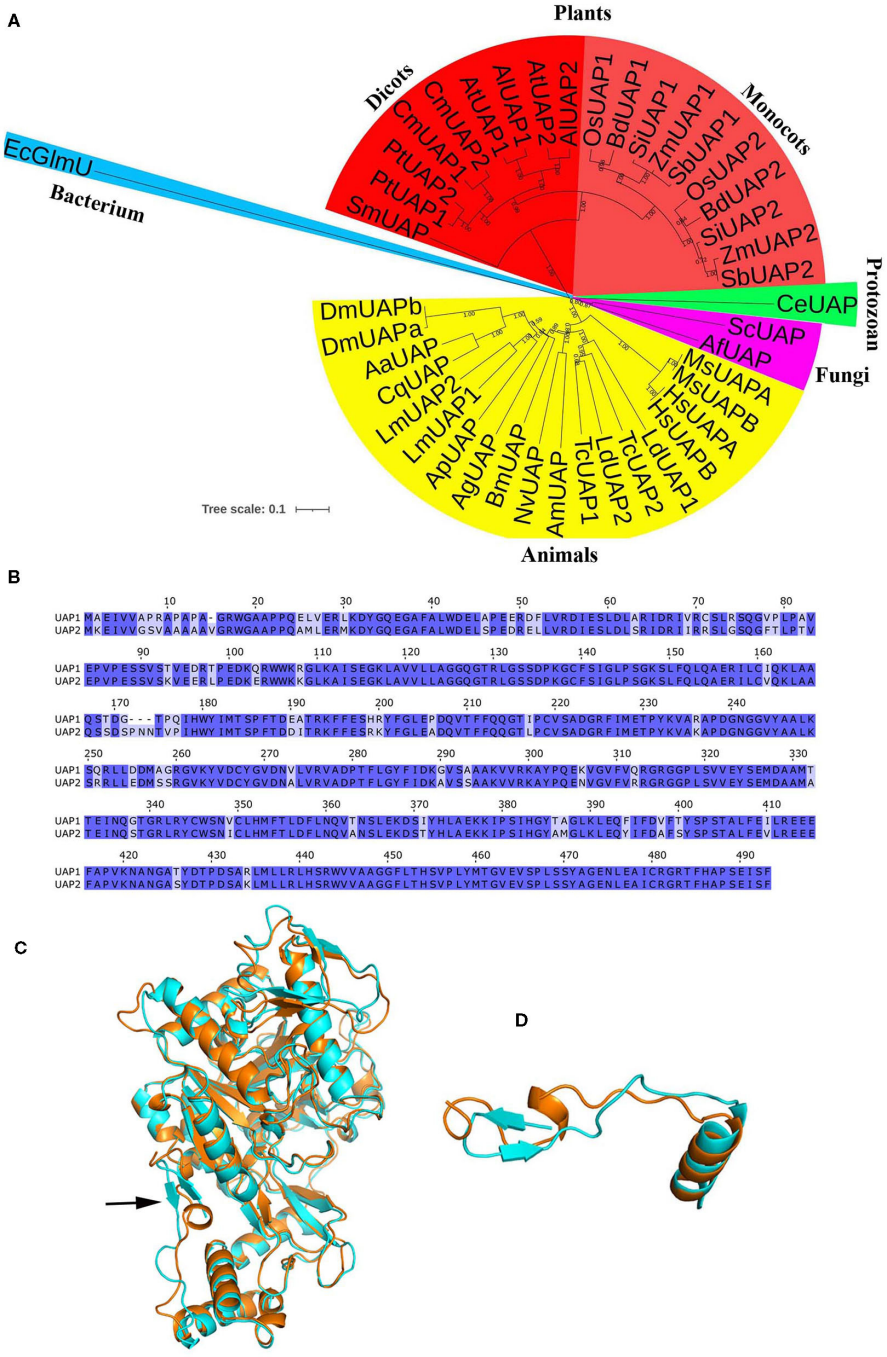
Bioinformatic analysis of UAP proteins. **(A)** Phylogenetic analysis of UAPs in different organisms. The tree was constructed based on the full-length amino acid sequences of UAPs. The tree scale indicates the units of amino acid substitutions per site. GenBank accession numbers are as follows: SmUAP, *Selaginella moellendorffii* (XP_024532253.1); PtUAP1, *Populus trichocarpa* (XP_006369047.1); PtUAP2, *Populus trichocarpa* (XP_002303345.3); CmUAP1, *Cucurbita moschata* (XP_022925908.1); CmUAP2, *Cucurbita moschata* (XP_022926514.1); AtUAP1, *Arabidopsis thaliana* (NP_564372.3); AlUAP1 *Arabidopsis lyrate* (XP_020870296.1); AtUAP2, *Arabidopsis thaliana* (NP_181047.1); AlUAP2, *Arabidopsis lyrate* (XP_002879530.1); OsUAP1, *Oryza sativa* (XP_015650402.1); BdUAP1, *Brachypodium distachyon* (XP_010234461.1); SiUAP1, *Setaria italica* (XP_004972591.1); ZmUAP1, *Zea mays* (PWZ43849.1); SbUAP1, *Sorghum bicolor* (XP_002444024.1); OsUAP2, *Oryza sativa* (XP_015633457.1); BdUAP2, *Brachypodium distachyon* (XP_003580539.1); SiUAP2, *Setaria italica* (XP_004976790.1); ZmUAP2, *Zea mays* (ONM14001.1); SbUAP2, *Sorghum bicolor* (XP_021317962.1); CeUAP, *Caenorhabditis elegans* (NP_497777.1); ScUAP, *Saccharomyces cerevisiae* (NP_010180.1); AfUAP, *Aspergillus fumigatus*, (XP_746714.1); MsUAPA, *Mus musculus* (NP_001291975.1, isoform A); MsUAPB, *Mus musculus* (NP_001291974.1, isoform B); HsUAPA, *Homo sapiens* (NP_001311044.1, isoform A); HsUAPB, *Homo sapiens* (NP_001311045.1, isoform B); LdUAP1, *Leptinotarsa decemlineata* (XP_023024179.1); LdUAP2, *Leptinotarsa decemlineata* (XP_023022882.1); TcUAP1, *Tribolium castaneum* (NP_001164533.1); TcUAP2, *Tribolium castaneum* (NP_001164534.1); AmUAP, *Apis mellifera* (XP_624349.1); NvUAP, *Nasonia vitripennis* (XP_001602623.1); BmUAP, *Bombyx mori* (AIQ85099.1); AgUAP, *Anopheles gambiae* (XP_317600.4); ApUAP, *Acyrthosiphon pisum* (XP_001944680.1); LmUAP1, *Locusta migratoria* (AGN56418.1); LmUAP2, *Locusta migratoria* (AGN56419.1); CqUAP, *Culex quinquefasciatus* (EDS38218.1); AaUAP, *Aedes aegypti* (EAT47260.1); DmUAPA, *Drosophila melanogaster* (NP_609032.1, isoform A); DmUAPB, *Drosophila melanogaster* (NP_723183.1, isoform B); EcGlmU, *Escherichia coli* (P0ACC7.1). **(B)** Alignment of the UAP1 and UAP2 protein sequences. **(C)** The 3D structure of the UAP1 and UAP2 proteins. Cyan, UAP1. Orange, UAP2. The arrow indicates the main difference between UAP1 and UAP2. **(D)** The structure of the first 20 amino acids of the two UAP proteins: The UAP1 has a β-sheet, while the UAP2 exhibits an α-helix.

To solve this problem, the evolutionary relationship of UAP1 protein was investigated. UAP proteins from diverse species, including plants, animals, fungi, and bacterium, were used to construct the NJ tree (Figure 2A). The results showed that UAP proteins were widely found in various organisms. However, the EcGlmU performing UAP functions in prokaryotes was significantly different from UAPs in eukaryotes. Besides, the UAP proteins were mainly divided into two clusters (plants and animals), indicating the different origins of UAPs from plants and animals. Interestingly, there exist two UAPs in some animals and most investigated plants, including rice. Through NCBI searching in the rice genome, a gene LOC_Os04g52370 is also annotated as UAP, thus named *UAP2*. The rice *UAP2* gene is highly homologous with the *UAP1* gene, separately sharing 82% identities for the coding sequence (Supplementary Figure 1) and 88% identities for the protein sequence (Figure 2B). The three-dimensional models of rice UAP2 and UAP1 proteins were generated using I-TASSER software. These two rice UAPs were predicted to have very similar 3D structures (Figure 2C) with some changes mainly at the N-terminal site (Figures 2C,D). In detail, for the first 20 amino acids of the two proteins, the UAP1 showed a β-sheet, while the UAP2 exhibited an α-helix (Figure 2D). Besides, the TM-score value of these two proteins was 0.97, which also indicated the high similarity on protein structures of UAP2 and UAP1. The analogous protein structures of UAP2 and UAP1 implied that these two proteins might have similar enzymatic functions. Presumably, the function of the *UAP2* gene would rescue the mutation of *uap1*, ensuring the normal growth of the young leaves of *uap1*.

**Figure 3:**
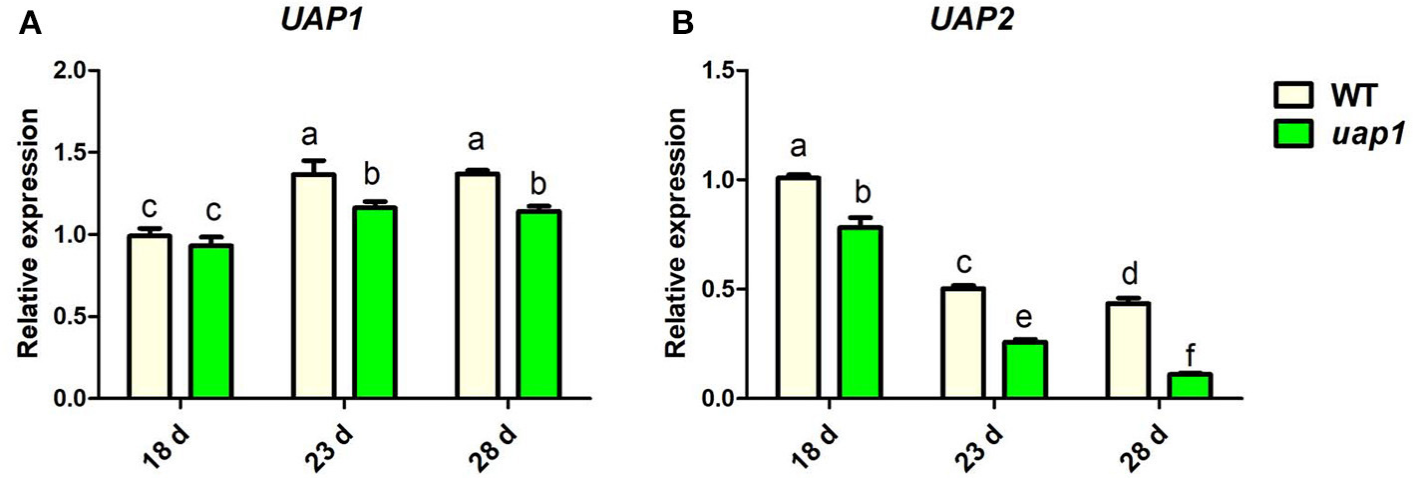
Relative expression of *UAP* genes in wild-type and *uap1* mutant leaves. **(A)** The *UAP1* gene. **(B)** The *UAP2* gene. Leaf samples in 18-, 23-, and 28-day-old plants at the seedling stage were detected (corresponding to Figure 1A). Relative expression of genes by qRT-PCR is normalized with reference genes *UBC, Profilin-2*, and *Actin1*. All data represent the mean ± *SD* of three biological replicates, and lowercase letters above columns indicate the statistically significant difference among all samples (one-way ANOVA).

To validate this hypothesis, the expression patterns of the *UAP2* gene and the *UAP1* gene were checked in wild-type and *uap1* plant leaves by qRT-PCR. The expression levels of the *UAP1* gene were constantly high and showed a rising trend separately in the 18-, 23-, 28-day-old wild-type and *uap1* leaves (Figure 3A). Meanwhile, the expression level of the *UAP2* gene was high in the 18-day-old wild-type and *uap1* leaves, but showed a declining trend in the 23- and 28-day-old wild-type and *uap1* leaves (Figure 3B). And the declining trend of the *UAP2* gene expression was more in *uap1* leaves than in wild-type leaves (Figure 3B). The fact that the reduction of expression of the *UAP2* gene in 23- and 28-day-old leaves of wild-type and *uap1* plants is perfectly synchronous with the appearance of defense response-related lesion-mimic spots and early senescence in the *uap1* mutant leaves. These results implied that the *UAP2* gene is highly possible to be responsible for the normal growth of the *uap1* young leaves.

### The Enzymatic Function of the UAP2 Protein Is Consistent With That of the UAP1 Protein

In order to identify the UAP2 protein performing the function of UAP, in common with the UAP1 protein, recombinant proteins of GST-UAP2 and GST-UAP1 were produced. The molecular weights of GST, UAP1, and UAP2 are theoretically 26, 54.071, and 54.447 kDa, respectively. GST, GST-UAP1 (about 80 kDa), and GST-UAP2 (about 80 kDa) were highly expressed after induction (Supplementary Figure 2, lanes 2–4). These three proteins were column-purified to detect the UAP enzymatic activity (Supplementary Figure 2, lanes 5–7).

**Figure 4:**
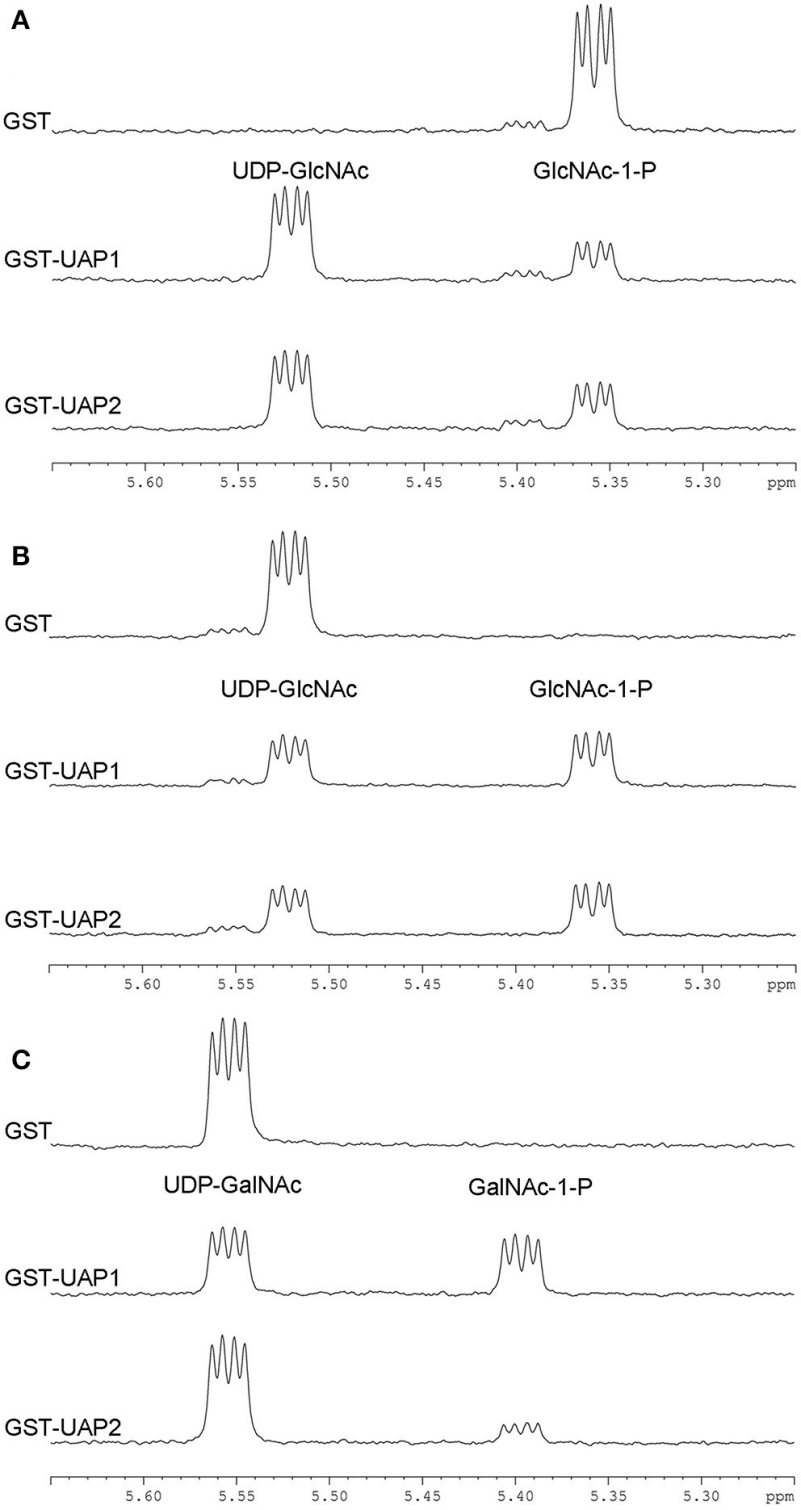
Enzymatic activities of UAP2 and UAP1 proteins based on ^1^H-NMR. **(A)** Forward UAP activity: UTP + GlcNAc-1-P → UDP-GlcNAc + PPi. The chemical shift (indicated by the “peak” shape) of 5.36 p.p.m is for the substrate GlcNAc-1-P and 5.52 p.p.m for the product UDP-GlcNAc. **(B)** Reverse UAP activity: UDP-GlcNAc + PPi → GlcNAc-1-P + UTP. The chemical shift of 5.52 p.p.m is for the substrate UDP-GlcNAc and 5.36 p.p.m for the product GlcNAc-1-P. **(C)** Reverse UAP activity: UDP-GalNAc + PPi → GalNAc-1-P + UTP. The chemical shift of 5.55 p.p.m is for the substrate UDP-GalNAc and 5.39 p.p.m for the product GalNAc-1-P. Data acquisition was performed at 1 h after the addition of the purified protein (GST, GST-UAP1, or GSP-UAP2) in the reaction mixture, with each line indicating each measurement. Results are representative of two independent experiments.

The enzymatic reaction of UAP2 and UAP1 proteins was monitored by ^1^H-NMR spectroscopy *in situ*. After 1-h enzymatic progression, forward conversion of GlcNAc-1-P (5.36 ppm) to UDP-GlcNAc (5.52 ppm) was observed both with GST-UAP2 and with GST-UAP1, but not with the GST control (Figure 4A). Similarly, the reverse conversion of UDP-GlcNAc (5.52 ppm) to GlcNAc-1-P (5.36 ppm) was both observed with GST-UAP2 and GST-UAP1, but not with GST (Figure 4B). GST-UAP2 and GST-UAP1 could also catalyze the reverse conversion of UDP-*N*-acetylgalactosamine (UDP-GalNAc) (5.55 ppm) to *N*-acetylgalactosamine-1-phosphate (GalNAc-1-P) (5.39 ppm) with the catalytic ability of GST-UAP2 weaker than GST-UAP1, whereas GST could not (Figure 4C). The forward reaction for the synthesis of UDP-GalNAc from GalNAc-1-P could not be tested since GalNAc-1-P was commercially unavailable.

These NMR-based assays provide unambiguous evidence that the UAP2 protein performs very similar UAP enzymatic activity with the UAP1 protein. It is speculated that UAP2 can compensate for the lost function of UAP1 in *uap1* plant leaves to maintain leaf survival from early senescence.

### The UAP2 Protein and the UAP1 Protein Located at the Same Subcellular Position to Perform Functions

To reveal where the UAP2 and UAP1 proteins perform their functions in the cells, the online SignalP, ChloroP, and TargetP servers were used to predict the subcellular localization of these two proteins. The SignalP found that the UAP1 and UAP2 proteins had no signal peptide. The ChloroP found that the UAP1 and UAP2 proteins didn't contain N-terminal chloroplast transit peptides. And the TargetP predicted that the UAP1 and UAP2 proteins were not localized in the chloroplast and mitochondria, but might be in any other subcellular place. According to these prediction results, it is speculated that the UAP1 and UAP2 proteins are most possibly localized in the cytoplasm.

**Figure 5:**
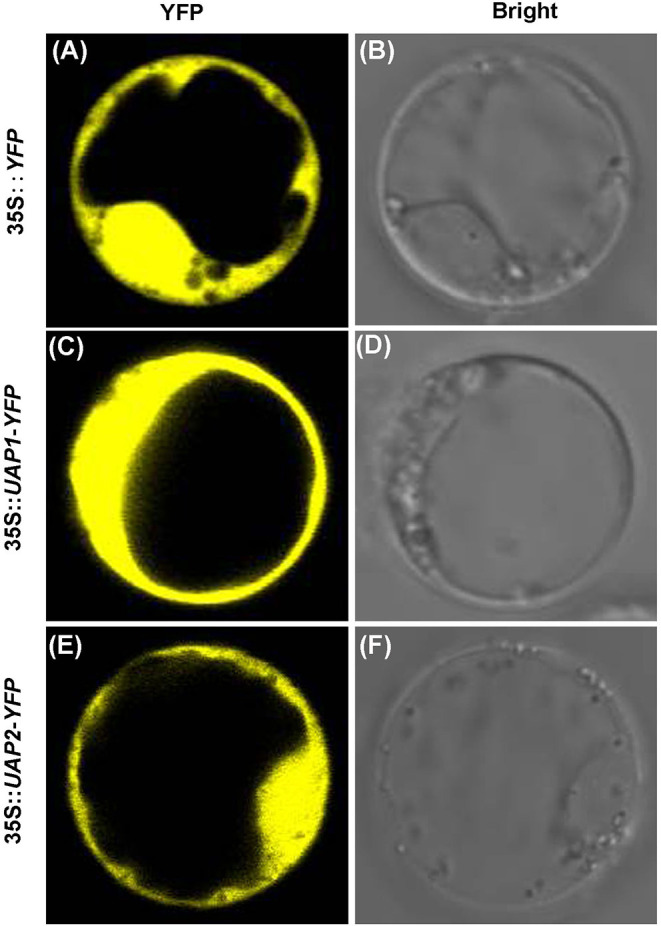
Subcellular localization of UAP1 and UAP2 proteins. Rice protoplast transformed with **(A,B)** empty vector 35S::*YFP* as control, **(C,D)** 35S::*UAP1*-*YFP*, and **(E,F)** 35S::*UAP2*-*YFP*. **(A,C,E)** YFP fluorescence images. **(B,D,F)** Bright field.

To find their real subcellular localization, the coding sequences of *UAP2* and *UAP1* were separately fused with *YFP*. Then the 35S::*UAP2*-*YFP*, 35S::*UAP1*-*YFP*, and 35S::*YFP* constructs were introduced into the rice protoplasts, respectively. Results showed that the YFP fluorescence signaling mechanisms indeed appeared throughout the cytoplasm in those protoplasts transformed with 35S::*UAP1*-*YFP* and 35S::*UAP2*-*YFP* (Figures 5C–F), like in protoplasts that were transformed with the control 35S::*YFP* (Figures 5A,B). These results indicated that the UAP2 and UAP1 proteins are both localized in the cytoplasm where they most presumably perform their functions.

### Overexpression of the *UAP2* Gene in *uap1* Plants Rescues Their Leaf Mutant Phenotype

**Figure 6:**
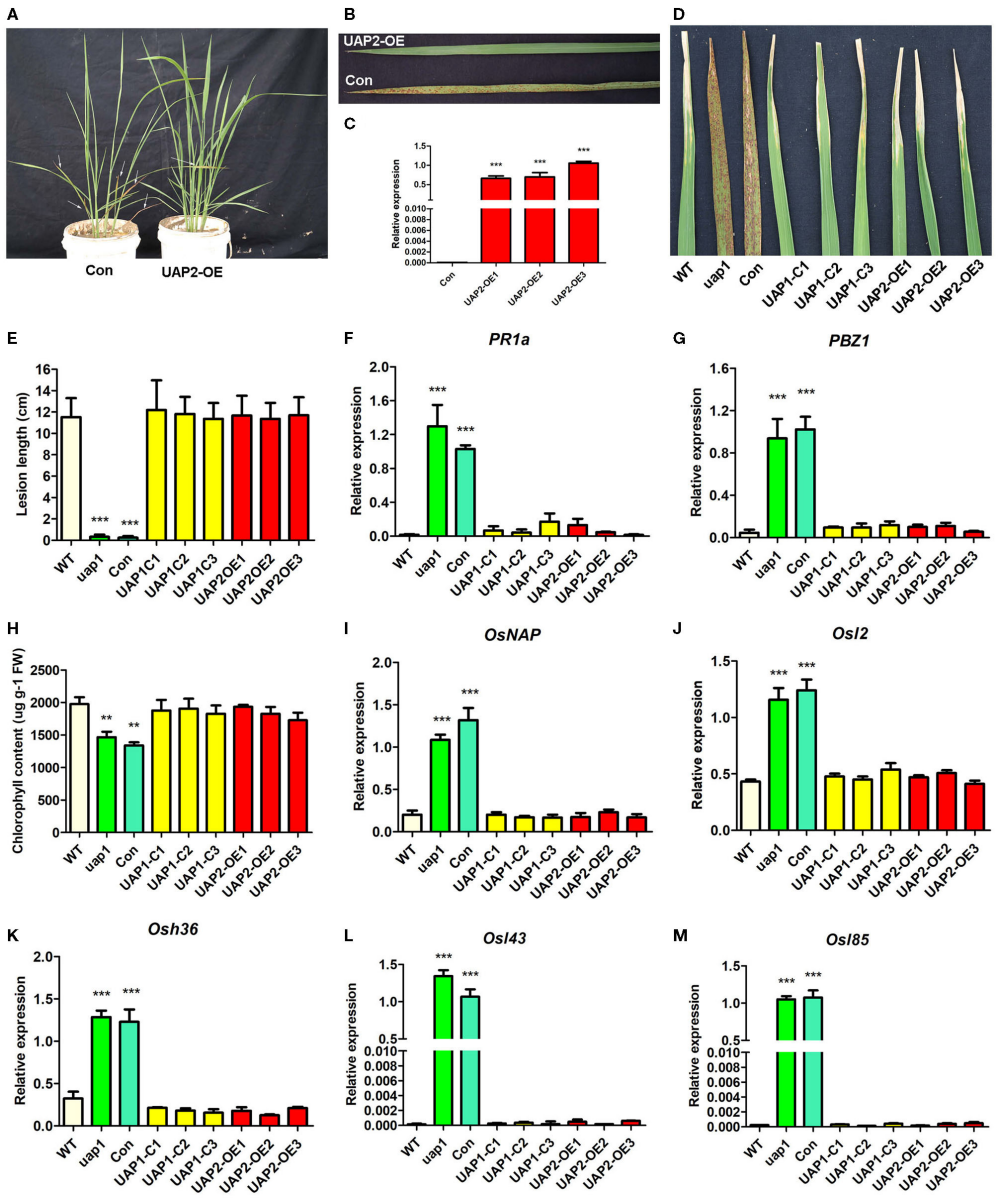
Phenotypic and molecular characteristics for transgenic plants with the *UAP2* gene rescuing the mutant phenotype of *uap1*. **(A)** Representative transgenic plants overexpressing *UAP2* (UAP2-OE) and with empty vector control (Con) at the tillering stage. Arrows indicate the leaves with the mutant phenotype. **(B)** Clear leaf phenotype in representative transgenic plants shown in **(A)**. **(C)** Relative expression of the *UAP2* gene in three representative transgenic plants overexpressing *UAP2* (UAP2-OE1, UAP2-OE2, and UAP2-OE3) and control transgenic plant with empty vector (Con). Data represent the mean ± *SD* of three biological replicates, and the asterisk indicates the statistically significant difference of overexpression samples compared with Con (***P < 0.0005, Student's *t*-test). **(D)** Infection phenotype after inoculation of plant leaves with bacterial blight pathogen PXO99. **(E)** Mean lesion length after inoculation of plant leaves with bacterial blight pathogen PXO99. Data represent the mean ± *SD* from three to five independent plants at tillering stage, and the asterisk indicates the statistically significant difference of samples compared with WT (***P < 0.0005, Student's *t*-test). **(F,G)** Relative expression of two defense signaling-related genes in plant leaves at tillering stage by qRT-PCR. **(H)** Chlorophyll contents in plant leaves at the tillering stage. FW: fresh weight. **(I–M)** Relative expression of senescence-associated transcription factors (*OsNAP*) and four *SAGs* (*Osl2, Osh36, Osl43*, and *Osl85*) in plant leaves at tillering stage. In **(F–M)**, data represent the mean ± *SD* of three biological replicates, and the asterisk indicates the statistically significant difference of samples compared with WT (**P < 0.005, ***P < 0.0005, Student's *t*-test). Relative expression of genes by qRT-PCR is normalized with reference genes *UBC, Profilin-2*, and *ARF*. WT, wild type. *uap1*, mutant of the *UAP1* gene. Con, transgenic plant with empty vector control. *UAP1*-C1, *UAP1*-C2, and *UAP1*-C3, three *uap1* transgenic lines with *UAP1* gene complementation. *UAP2*-OE1, *UAP2*-OE2, and *UAP2*-OE3, three *uap1* transgenic lines overexpressing the *UAP2* gene.

In order to identify the role of the *UAP2* gene in compensating for the lost function of the *UAP1* gene in *uap1* mutant plants, a transgenic experiment to overexpress the *UAP2* gene in the *uap1* plants was performed. The full-length CDS fragment (1,482 bp) of *UAP2* was constructed into the overexpression vector p*UAP2*-OE using the ubiquitin promoter. The p*UAP2*-OE vector and the empty control vector were transferred into the *uap1* calli by *A. tumefaciens*-mediated transformation. Eight independent transgenic lines overexpressing the *UAP2* gene were obtained, showing a complete rescue of the mutant phenotype; meanwhile, six independent transgenic lines with the empty vector were obtained, failing to compensate the *uap1* mutant (Figures 6A,B). The expression changes of the *UAP2* gene in three representative transgenic lines with *UAP2* overexpression were additionally detected. The result showed that the *UAP2* gene was overexpressed significantly in these three transgenic lines (Figure 6C), which were thus used for subsequent analysis.

The defense response for plant resistance was tested in leaves of wild-type plants, *uap1* plants, *uap1* transgenic plants with the empty vector, *uap1* transgenic lines with the *UAP1* gene complementary vector, and *uap1* transgenic lines with the *UAP2* gene overexpression vector. To test their resistance to the pathogen, these plants were inoculated with the bacterial blight strain PXO99 at the tillering stage. The *uap1* plants and *uap1* transgenic plants with the empty vector exhibited significantly enhanced resistance, while three *uap1* transgenic lines overexpressing the *UAP2* gene showed the typical response to bacterial blight diseases, like as the wild-type plants and three *uap1* transgenic lines with *UAP1* gene complementation (Figures 6D,E). Correspondingly, expression levels of two defense response genes (*PR1a* and *PBZ1*) were analyzed by qRT-PCR. Results showed that expression levels of *PR1a* and *PBZ1* were all up-regulated in leaves of *uap1* plants and *uap1* transgenic plants with the empty vector compared with the wild type, but recovered to the normal level in three *uap1* transgenic lines overexpressing the *UAP2* gene, as these three *uap1* transgenic lines with *UAP1* gene complementation (Figures 6F,G). These phenotypic and molecular studies all supported the fact that overexpression of the *UAP2* gene in *uap1* can rescue its defense response phenotype, just the same as the *UAP1* gene, implying the similar gene function of these two *UAP* genes on plant defense.

Chlorophyll contents were measured in leaves of wild-type plants, *uap1* plants, *uap1* transgenic plants with the empty vector, *uap1* transgenic lines with the *UAP1* gene complementary vector, and *uap1* transgenic lines with the *UAP2* gene overexpression vector. Results showed that the chlorophyll contents in leaves of three *uap1* transgenic lines overexpressing the *UAP2* gene were restored to the normal level, as in leaves of wild-type plants and three *uap1* transgenic lines with *UAP1* gene complementation, while those in leaves of *uap1* plants and *uap1* transgenic plants with the empty vector were significantly decreased (Figure 6H). Expression levels of the senescence-associated transcription factor *OsNAP* and four SAGs (*Osl2, Osh36, Osl43*, and *Osl85*) were additionally detected by qRT-PCR in these materials. Results suggested that these five genes were equally expressed in the leaves of wild-type plants, *uap1* transgenic lines overexpressing the *UAP2* gene, and *uap1* transgenic lines with *UAP1* gene complementation, but they were up-regulated in leaves of *uap1* plants and *uap1* transgenic plants with the empty vector (Figures 6I–M). These physiological and molecular studies all supported the fact that overexpression of the *UAP2* gene in *uap1* can rescue its early leaf senescence phenotype, just as the *UAP1* gene, indicating the similar function of these two *UAP* genes on leaf senescence.

Taken together, phenotypes of defense response-related leaf spots and subsequent early leaf senescence of the *uap1* mutant can also be rescued by the *UAP2* gene, in addition to the *UAP1* gene, providing effective results for the synergetic role of *UAP2* and *UAP1* genes on protecting leaf from early senescence.

### The *UAP2* Gene Is Mainly Expressed in Young Leaves, While the *UAP1* Gene Maintains Continuous High Expression During the Whole Leaf Development

**Figure 7:**
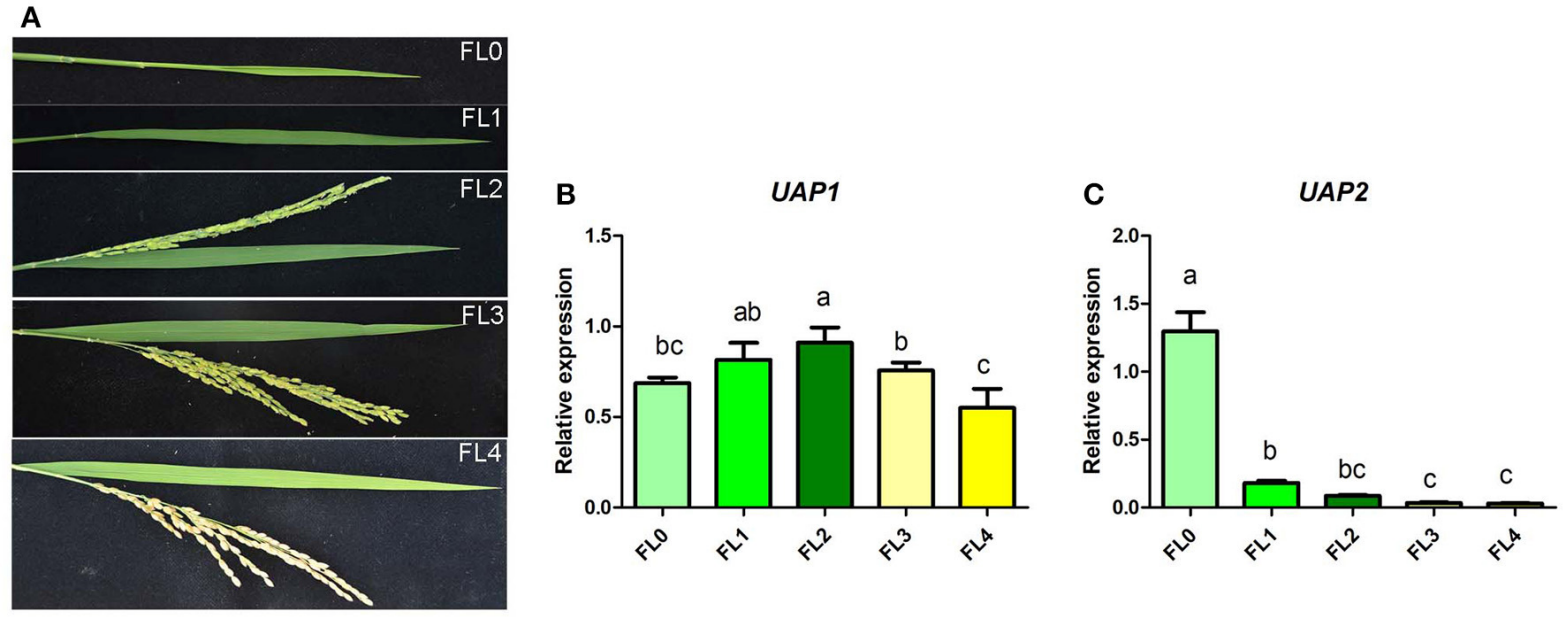
Relative expression of the *UAP1* and *UAP2* genes during flag leaf development. **(A)** Flag leaf phenotype at unexpanded young leaf stage (FL0), booting stage (FL1), flowering stage (FL2), filling stage (FL3), and maturation stage (FL4). **(B,C)** Relative expression levels of the *UAP1* and *UAP2* genes. Data represent the mean ± *SD* of three biological replicates, and lowercase letters above columns indicate the statistically significant difference among all samples (one-way ANOVA). Relative expression of genes by qRT-PCR is normalized with reference genes *UBC, Profilin-2*, and *ARF*.

The expression profile of the *UAP2* and *UAP1* genes was identified in rice flag leaves during their whole developmental periods, including unexpanded young leaf stage, booting stage, flowering stage, filling stage, and maturation stage (Figure 7A). Results showed that the expression level of the *UAP1* gene slightly increased in flag leaf from the unexpanded young leaf stage to flowering stage, and then showed a minor decrease at filling stage and maturation stage (Figure 7B). As a whole, the *UAP1* gene is expressed at a continuously high level in flag leaves during all these developmental stages. Meanwhile, the expression level of the *UAP2* gene was the highest at the unexpanded young leaf stage, but soon exhibited a sharp and continuous decline at the next four stages (Figure 7C). These results implied the fact that the *UAP2* gene mainly plays a role at the young leaf stage, while the *UAP1* gene performs its role during the whole leaf developmental stages.

## Discussion

### *UAP* Genes Are Essential for Survival Among Organisms

UDP-*N*-acetylglucosamine pyrophosphorylase catalyzes the final step of the hexosamine biosynthetic pathway, producing UDP-GlcNAc, an essential sugar moiety involved in protein glycosylation, glycolipids, and GPI-anchor-linked protein (Raetz and Whitfield, 2002; Hancock, 2004; Stanley et al., 2015). UDP-*N*-acetylglucosamine pyrophosphorylases are conserved and widely distributed among organisms (Figure 2A). Their functions have been partially studied from prokaryotes to eukaryotes, such as bacteria, fungi, animals, and plants. As reported, the copy number of the *UAP* gene varies depending on the species. In fungi like yeast and *A. fumigatus, UAP* is a single gene, showing the essential roles in cell morphogenesis and survival (Mio et al., 1998; Fang et al., 2013). In *T. brucei*, the single *UAP* gene is also absolutely necessary for cell growth, and its null mutant will cause the happening of cell lysis (Stokes et al., 2008). For the insects, there were two *UAP* genes reported in *T. castaneum, Locusta migratoria*, and *Leptinotarsa decemlineata*, but only a single *UAP* gene in most other investigated insects, such as *Aedes aegypti, Culex quinquefasciatus, D. melanogaster, Bombyx mori, Anopheles gambiae, Acyrthosiphon pisum, Apis mellifera*, and *Nasonia vitripennis* (Arakane et al., 2011; Liu et al., 2013; Shi et al., 2016). Both *UAP1* and *UAP2* were found to be critical for individual development and survival in *T. castaneum* and *Leptinotarsa decemlineata*, while only *UAP1* was identified as essential for the development and survival of *Locusta migratoria* at least in nymphal stage (Arakane et al., 2011; Liu et al., 2013; Shi et al., 2016). The humans only have a single *UAP* gene, but two isoforms, *HsUAPA* and *HsUAPB* (also called *AGX1* and *AGX2*), which can use GlcNAc-1-P or *N*-acetylgalactosamine-1-phosphate (GalNAc-1-P) as substrates to synthesize UDP-GlcNAc or UDP-GalNAc, with the preferred substrate of GlcNAc-1-P for UAPA and the preferred substrate of GalNAc-1-P for UAPB (Wang-Gillam et al., 1998; Peneff et al., 2001). The same situation of one *UAP* gene with two isoforms is found in the mammal *Mus musculus* (Figure 2A). No *UAP* mutant was reported in these two higher model animals; however, *UAP1* was found to be overexpressed in prostate cancer and protect against inhibitors of *N*-linked glycosylation, conferring a growth advantage (Itkonen et al., 2015). It is interesting to find that two *UAP* genes are found in the analyzed monocotyledons and dicotyledons (Figure 2A). The single gene mutants of *UAP1* and *UAP2* both showed no obvious phenotype in *Arabidopsis*, but their homozygous double mutant was lethal, reflecting the functional redundancy of these two genes in survival of *Arabidopsis* plants (Chen et al., 2014). In summary, the *UAP* genes play an essential role in the survival of cells or individuals for different organisms.

### Possible Mechanisms by Which *UAP2* Cooperates With *UAP1* on Protecting Leaves From Lesion-Mimic Spots and Subsequent Early Senescence

The mutation of the *UAP1* gene in rice has lost its protein enzymatic function, leading to the appearance of phenotypes of lesion-mimic spots and early senescence in *uap1* (also called *spl29*) mutant leaves (Wang et al., 2015). However, it is interesting that every newly grown leaf of *uap1* could normally grow for a period of time, and then the defense response-related lesion-mimic spots and early leaf senescence appeared and became aggravated with the increase in growth time (Figure 1A). The UAP enzymatic function of the uap1 protein was found lost due to the substitution of a key single amino acid (Wang et al., 2015). This meant that the *uap1* mutant leaves lack the UAP1 enzymatic activity and there probably exists another enzyme compensating it.

A bioinformatic search on the rice genome database identified the *UAP2* gene, the homologous gene of *UAP1*. The two rice *UAP* genes are located on different chromosomes and controlled by different promoters. The *UAP2* and *UAP1* genes showed high homology for the nucleotide and protein sequences (Supplementary Figure 1 and Figure 2B), implying that these two genes must have derived from a recent gene duplication event in rice. Transcriptional expression of the *UAP2* gene was high when the newly grown leaves of *uap1* were normal, but decreased a lot accompanying with the appearance and exacerbation of lesion-mimic sports and early leaf senescence (Figure 3B). It is seemed that the dosage effect and time specificity of the *UAP2* gene expression can influence the leaf cell death of the *uap1* mutant. Thus, we speculated that the high expression of the *UAP2* gene can compensate the lost function of the *UAP1* gene in *uap1* young leaves and that during the process of the leaf development to mature, the *UAP2* gene expression decreases and the function of total UAPs in the *uap1* leaf cells is not enough to maintain the normal growth, producing defense response-related lesion-mimic spots and early leaf senescence. In this study, the enzymatic assay verified that the UAP2 protein performed a very similar or overlapping UAP enzymatic activity with the UAP1 protein (Figure 4). And the UAP2 protein showed the same subcellular localization as the UAP1 protein (Figure 5), meaning that the UAP2 and UAP1 proteins perform their enzymatic reactions in the same cellular location. These molecular studies predominantly suggested that the *UAP2* gene was able to compensate for the lost function of the *UAP1* gene in *uap1* mutant. Eventually, the transgene of *UAP2* into *uap1* mutant recovered its leaf mutant phenotypes (Figure 6), providing direct evidence that the *UAP2* gene can perform similar biological functions as the *UAP1* gene, protecting the *uap1* mutant from lesion-mimic sports and subsequent early leaf senescence. In addition, transcriptional expression of the *UAP2* gene was high in young flag leaf, but decreased with the leaf becoming mature, while expression levels of the *UAP1* gene were continuously high during all analyzed leaf developmental stages (Figure 7), suggesting that the *UAP2* gene mainly functioned in young leaves, and the *UAP1* gene functioned in all leaf stages.

### The Possible Role of Protein Glycosylation on Cell Death or Senescence

Protein glycosylation is essential for the proper folding, targeting, and functioning of proteins. So far, several studies have also been reported to reveal the glycosylation being involved in plant defense, senescence, and cell death. The *Arabidopsis* glucosyltransferase UGT76B1 conjugates isoleucic acid and modulates plant defense and senescence by small-molecule glucosylation (von Saint Paul et al., 2011). The rice OsDGL1, a homolog of an oligosaccharyltransferase complex subunit, is involved in *N*-glycosylation and cell death in the root (Qin et al., 2013). The *N*-acetylglucosaminyltrasferase I (GnT1) mutant exhibited complete inhibition of *N*-glycan maturation, resulting in early lethality without transition to the reproductive stage in rice (Fanata et al., 2013). The rice *PLS2*, encoding a glycosyltransferase, its mutation makes premature leaf senescence begin at the tillering stage (Wang et al., 2018). Interestingly, a qualitative analysis of *N*-linked glycoproteome in the senescent flag leaf of rice has identified 183 *N*-glycoproteins involved in various and famous senescence-related biological processes (Huang et al., 2019).

*N*-Linked glycans are the components of most membrane-associated and secreted proteins in eukaryotic cells. And UDP-GlcNAc is an initial and key sugar donor of *N*-glycan synthesis for glycosylation. The *GNA1* encodes the glucosamine-6-phosphate acetyltransferase in the pathway for the biosynthesis of UDP-GlcNAc. And the *gna1* mutants in *Arabidopsis* and rice showed temperature-sensitive growth defects of the root, accompanying with insufficient biosynthesis of endogenous UDP-GlcNAc and impairment of protein *N*-glycosylation (Jiang et al., 2005; Nozaki et al., 2012). In rice, UAP1 is the very enzyme for the catalytic synthesis of UDP-GlcNAc, and functional inactivation of UAP1 induces early leaf senescence and defense responses (Wang et al., 2015). In *Arabidopsis*, GlcNAc1pUT-1 and GlcNAc1pUT-2 catalyze the biosynthesis of UDP-GlcNAc (Yang et al., 2010). The single mutants *glcna.ut1* and *glcna.ut2* revealed no obvious phenotype but their homozygous double mutant was lethal, revealing the GlcNAc1pUTs' indispensable role in the unique mediation of gametogenesis and embryogenesis, despite the overlapping functions (Chen et al., 2014). Taking together, the synthesis defect for UDP-GlcNAc leads to cell death in different plant tissues, probably attributing to the divergent demand for UDP-GlcNAc contents in these tissues to sustain normal cell survival. In this study, the UAP2 protein is found to be able to synthesize UDP-GlcNAc, just as the UAP1 protein does (Figure 4A). Meanwhile, the UAP1 and UAP2 proteins are both localized in the cytoplasm in rice (Figure 5), where they can function to synthesize UDP-GlcNAc, and this is coincident with the fact that GlcNAc is used for the *N*-glycan biosynthesis on the cytosolic side of the endoplasmic reticulum (ER) (Stanley et al., 2015). It is speculated that the dosage defect of UDP-GlcNAc and subsequently induced abnormal of protein glycosylation are responsible for the mutant phenotypes of *uap1*. Although there is no direct evidence linking UDP-GlcNAc with the lesion-mimic spots and early leaf senescence phenotypes found in *uap1* mutants, it will be interesting to study the UDP-GlcNAc levels, protein glycosylation status, and the downstream molecular pathways in the future, to better reveal the biological roles of the *UAP* proteins in leaf survival.

## Conclusion

Our data demonstrate that *UAP2*, the homologous gene of *UAP1*, could maintain the short period's normal growth of the *uap1* mutant leaves. The expression level of the *UAP2* gene was high in the initial normal growth stage, but decreased accompanying with the appearance of defense response-related lesion-mimic spots and early senescence of the *uap1* mutant leaves. The UAP2 protein performed a very similar UAP enzymatic activity with the UAP1 protein. And these two UAP proteins were both localized in the cytoplasm to perform their function. Overexpression of the *UAP2* gene in the *uap1* mutant could rescue its mutant phenotype, confirming the similar molecular and biological function of the *UAP2* gene with the *UAP1* gene. The *UAP2* gene was mainly expressed in the young leaves, while the *UAP1* gene maintains continuous high expression during the whole leaf development. Taking together, rice UAP2 cooperates with UAP1 to perform a synergetic function for leaf survival during its developmental process, protecting the leaf from early senescence. However, further investigation is required to elucidate the downstream pathways underlying rice *UAPs*.

## Data Availability Statement

The original contributions presented in the study are included in the article/Supplementary Materials, further inquiries can be directed to the corresponding author/s.

## Author Contributions

ZW conceptualized this study research and wrote the manuscript. QW and LW did experiments for transgenic plants. YS helped in the data analysis. TL helped in language revision. KH performed qRT-PCR for *UAP* genes in flag leaves. SL and HZho performed bioinformatic analysis. HZha and JL helped in bioinformatic analysis and manuscript revision. YL helped in molecular experiments. YH helped in data consolidation and manuscript revision. All authors contributed to the article and approved the submitted version.

## Conflict of Interest

The authors declare that the research was conducted in the absence of any commercial or financial relationships that could be construed as a potential conflict of interest.

## Supplementary Material

Supplementary Figure 1 Alignment of the coding sequences of the *UAP1* and *UAP2* genes.

Supplementary Figure 2 SDS/PAGE of proteins. Lane 1, prestained protein ladder. Total soluble proteins from *E.coli* cells expressing control empty vector (lane 2), recombinant *UAP1* (lane 3), and recombinant *UAP2* (lane 4). Purified GST (lane 5), GST-UAP1 (lane 6), and GST-UAP2 (lane 7) proteins. Bands of GST, GST-UAP1, and GST-UAP2 are indicated by *arrows*.

Supplementary Table 1 All primers used for qRT-PCR analysis.

Supplementary Table 2 Primers for vector construction and confirmation of positive transgenic plants.

## References

[B1] Arakane Y. Baguinon M. C. Jasrapuria S. Chaudhari S. Doyungan A. Kramer K. J.. (2011). Both UDP N-acetylglucosamine pyrophosphorylases of *Tribolium castaneum* are critical for molting, survival and fecundity. Insect Biochem. Mol. Biol. 41, 42–50. 10.1016/j.ibmb.2010.09.011 20920581

[B2] Chen Y. H. Shen H. L. Hsu P. J. Hwang S. G. Cheng W. H. (2014). N-acetylglucosamine-1-P uridylyltransferase 1 and 2 are required for gametogenesis and embryo development in *Arabidopsis thaliana*. Plant Cell Physiol. 55, 1977–1993. 10.1093/pcp/pcu127 25231969

[B3] Fanata W. I. Lee K. H. Son B. H. Yoo J. Y. Harmoko R. Ko K. S.. (2013). N-glycan maturation is crucial for cytokinin-mediated development and cellulose synthesis in *Oryza sativa*. Plant J. 73, 966–979. 10.1111/tpj.12087 23199012

[B4] Fang W. Du T. Raimi O. G. Hurtado-Guerrero R. Urbaniak M. D. Ibrahim A. F.. (2013). Genetic and structural validation of *Aspergillus fumigatus* UDP-N-acetylglucosamine pyrophosphorylase as an antifungal target. Mol. Microbiol. 89, 479–493. 10.1111/mmi.12290 23750903 PMC3888555

[B5] Hancock J. F. (2004). GPI-anchor synthesis: Ras takes charge. Dev Cell 6, 743–745. 10.1016/j.devcel.2004.05.011 15177021

[B6] Huang X. Zhang H. Liao J. Wei L. Guo R. Xiao W.. (2019). Qualitative analysis of N-linked glycoproteome in senescent flag leaf of rice. Plant Growth Regul. 88, 309–326. 10.1007/s10725-019-00509-y

[B7] Itkonen H. M. Engedal N. Babaie E. Luhr M. Guldvik I. J. Minner S.. (2015). UAP1 is overexpressed in prostate cancer and is protective against inhibitors of N-linked glycosylation. Oncogene 34, 3744–3750. 10.1038/onc.2014.307 25241896

[B8] Jiang H. Wang S. Dang L. Wang S. Chen H. Wu Y.. (2005). A novel short-root gene encodes a glucosamine-6-phosphate acetyltransferase required for maintaining normal root cell shape in rice. Plant Physiol. 138, 232–242. 10.1104/pp.104.058248 15849305 PMC1104178

[B9] Katoh K. Standley D. M. (2013). MAFFT multiple sequence alignment software version 7: improvements in performance and usability. Mol. Biol. Evol. 30, 772–780. 10.1093/molbev/mst010 23329690 PMC3603318

[B10] Lee R. H. Wang C. H. Huang L. T. Chen S. C. (2001). Leaf senescence in rice plants: cloning and characterization of senescence up-regulated genes. J. Exp. Bot. 52, 1117–1121. 10.1093/jexbot/52.358.1117 11432928

[B11] Liang C. Wang Y. Zhu Y. Tang J. Hu B. Liu L.. (2014). OsNAP connects abscisic acid and leaf senescence by fine-tuning abscisic acid biosynthesis and directly targeting senescence-associated genes in rice. Proc. Natl. Acad. Sci. U.S.A. 111, 10013–10018. 10.1073/pnas.1321568111 24951508 PMC4103337

[B12] Liu X. Li F. Li D. Ma E. Zhang W. Zhu K. Y.. (2013). Molecular and functional analysis of UDP-N-acetylglucosamine pyrophosphorylases from the migratory locust, *Locusta migratoria*. PLoS ONE 8:e71970. 10.1371/journal.pone.0071970 23977188 PMC3747057

[B13] Lorrain S. Vailleau F. Balague C. Roby D. (2003). Lesion mimic mutants: keys for deciphering cell death and defense pathways in plants? Trends Plant Sci. 8, 263–271. 10.1016/S1360-1385(03)00108-0 12818660

[B14] Mengin-Lecreulx D. van Heijenoort J. (1993). Identification of the glmU gene encoding N-acetylglucosamine-1-phosphate uridyltransferase in *Escherichia coli*. J. Bacteriol. 175, 6150–6157. 10.1128/jb.175.19.6150-6157.1993 8407787 PMC206709

[B15] Mio T. Yabe T. Arisawa M. Yamada-Okabe H. (1998). The eukaryotic UDP-N-acetylglucosamine pyrophosphorylases. Gene cloning, protein expression, and catalytic mechanism. J. Biol. Chem. 273, 14392–14397. 10.1074/jbc.273.23.14392 9603950

[B16] Nozaki M. Sugiyama M. Duan J. Uematsu H. Genda T. Sato Y. (2012). A missense mutation in the glucosamine-6-phosphate N-acetyltransferase-encoding gene causes temperature-dependent growth defects and ectopic lignin deposition in Arabidopsis. Plant Cell 24, 3366–3379. 10.1105/tpc.112.102806 22932674 PMC3462637

[B17] Peneff C. Ferrari P. Charrier V. Taburet Y. Monnier C. Zamboni V.. (2001). Crystal structures of two human pyrophosphorylase isoforms in complexes with UDPGlc(Gal)NAc: role of the alternatively spliced insert in the enzyme oligomeric assembly and active site architecture. EMBO J. 20, 6191–6202. 10.1093/emboj/20.22.6191 11707391 PMC125729

[B18] Qin C. Li Y. Gan J. Wang W. Zhang H. Liu Y.. (2013). OsDGL1, a homolog of an oligosaccharyltransferase complex subunit, is involved in N-glycosylation and root development in rice. Plant Cell Physiol. 54, 129–137. 10.1093/pcp/pcs159 23220823

[B19] Raetz C. R. Whitfield C. (2002). Lipopolysaccharide endotoxins. Annu. Rev. Biochem. 71, 635–700. 10.1146/annurev.biochem.71.110601.135414 12045108 PMC2569852

[B20] Schimmelpfeng K. Strunk M. Stork T. Klambt C. (2006). Mummy encodes an UDP-N-acetylglucosamine-dipohosphorylase and is required during Drosophila dorsal closure and nervous system development. Mech. Dev. 123, 487–499. 10.1016/j.mod.2006.03.004 16793242

[B21] Shi J. F. Fu J. Mu L. L. Guo W. C. Li G. Q. (2016). Two *Leptinotarsa uridine* diphosphate N-acetylglucosamine pyrophosphorylases are specialized for chitin synthesis in larval epidermal cuticle and midgut peritrophic matrix. Insect Biochem. Mol. Biol. 68, 1–12. 10.1016/j.ibmb.2015.11.005 26592348

[B22] Stanley P. Taniguchi N. Aebi M. (2015). “N-Glycans,” in Essentials of Glycobiology, eds A. Varki, R. D. Cummings, J. D. Esko, P. Stanley, G. W. Hart, M. Aebi, A. G. Darvill, T. Kinoshita, N. H. Packer, J. H. Prestegard, R. L. Schnaar and P. H. Seeberger (Cold Spring Harbor, NY: Cold Spring Harbor Laboratory Press), 99–111. 27010055

[B23] Stokes M. J. Guther M. L. Turnock D. C. Prescott A. R. Martin K. L. Alphey M. S.. (2008). The synthesis of UDP-N-acetylglucosamine is essential for bloodstream form *Trypanosoma brucei in vitro* and *in vivo* and UDP-N-acetylglucosamine starvation reveals a hierarchy in parasite protein glycosylation. J. Biol. Chem. 283, 16147–16161. 10.1074/jbc.M709581200 18381290 PMC2414269

[B24] von Saint Paul V. Zhang W. Kanawati B. Geist B. Faus-Kessler T. Schmitt-Kopplin P.. (2011). The Arabidopsis glucosyltransferase UGT76B1 conjugates isoleucic acid and modulates plant defense and senescence. Plant Cell 23, 4124–4145. 10.1105/tpc.111.088443 22080599 PMC3246326

[B25] Wang M. Zhang T. Peng H. Luo S. Tan J. Jiang K.. (2018). Rice premature leaf senescence 2, encoding a Glycosyltransferase (GT), is involved in leaf senescence. Front. Plant Sci. 9:560. 10.3389/fpls.2018.00560 29755498 PMC5932172

[B26] Wang Z. Wang Y. Hong X. Hu D. Liu C. Yang J.. (2015). Functional inactivation of UDP-N-acetylglucosamine pyrophosphorylase 1 (UAP1) induces early leaf senescence and defense responses in rice. J. Exp. Bot. 66, 973–987. 10.1093/jxb/eru456 25399020 PMC4321554

[B27] Wang Z. Wang Y. Yang J. Hu K. An B. Deng X.. (2016). Reliable selection and holistic stability evaluation of reference genes for rice under 22 different experimental conditions. Appl. Biochem. Biotechnol. 179, 753–775. 10.1007/s12010-016-2029-4 26940571

[B28] Wang-Gillam A. Pastuszak I. Elbein A. D. (1998). A 17-amino acid insert changes UDP-N-acetylhexosamine pyrophosphorylase specificity from UDP-GalNAc to UDP-GlcNAc. J. Biol. Chem. 273, 27055–27057. 9765219 10.1074/jbc.273.42.27055

[B29] Waterhouse A. M. Procter J. B. Martin D. M. Clamp M. Barton G. J. (2009). Jalview Version 2–a multiple sequence alignment editor and analysis workbench. Bioinformatics 25, 1189–1191. 10.1093/bioinformatics/btp033 19151095 PMC2672624

[B30] Yang J. Yan R. Roy A. Xu D. Poisson J. Zhang Y. (2015). The I-TASSER Suite: protein structure and function prediction. Nat Methods 12, 7–8. 10.1038/nmeth.3213 25549265 PMC4428668

[B31] Yang T. Echols M. Martin A. Bar-Peled M. (2010). Identification and characterization of a strict and a promiscuous N-acetylglucosamine-1-P uridylyltransferase in Arabidopsis. Biochem. J. 430, 275–284. 10.1042/BJ20100315 20557289

[B32] Yu C. Wang L. Chen C. He C. Hu J. Zhu Y.. (2014). Protoplast: a more efficient system to study nucleo-cytoplasmic interactions. Biochem. Biophys. Res. Commun. 450, 1575–1580. 10.1016/j.bbrc.2014.07.043 25026554

[B33] Zhang J. Zhang Y. Du Y. Chen S. Tang H. (2011). Dynamic metabonomic responses of tobacco (*Nicotiana tabacum*) plants to salt stress. J. Proteome Res. 10, 1904–1914. 10.1021/pr101140n 21323351

[B34] Zhang W. Jones V. C. Scherman M. S. Mahapatra S. Crick D. Bhamidi S.. (2008). Expression, essentiality, and a microtiter plate assay for mycobacterial GlmU, the bifunctional glucosamine-1-phosphate acetyltransferase and N-acetylglucosamine-1-phosphate uridyltransferase. Int. J. Biochem. Cell Biol. 40, 2560–2571. 10.1016/j.biocel.2008.05.003 18573680 PMC2602953

[B35] Zhang Y. Skolnick J. (2005). TM-align: a protein structure alignment algorithm based on the TM-score. Nucleic Acids Res. 33, 2302–2309. 10.1093/nar/gki524 15849316 PMC1084323

