# Supplementary material for: UDP-N-Acetylglucosamine Pyrophosphorylase 2 (UAP2) and 1 (UAP1) Perform Synergetic Functions for Leaf Survival in Rice
Source: Front Plant Sci. 2021 Jun 24;12:685102. doi: 10.3389/fpls.2021.685102 (PMC8264299; doi:10.3389/fpls.2021.685102)
Supplement: Supplementary Figure 1 — Alignment of the coding sequences of the UAP1 and UAP2 genes. [file Image_1.PDF]

```
UAP1    1 ATGGCGGAGATCGTGGTGGCGCCGCGGGCGCCGGCGCCGGCG---GGGAGGTGGGGCGCCGCGCCGCCGCAGGAGCTGGTCGAGAGGCTCAAGGACTACGGCCAG  102
UAP2    1 ATGAAGGAGATAGTGGTTGGGTCGGTGGCGGCGGCGGCGGCGGTGGGGAGGTGGGGAGCGGCGCCGCCGCAGGCGATGCTGGAGAGGATGAAGGATTACGGGCAG  105

UAP1  103 GAGGGCGCCTTCGCGCTCTGGGACGAGCTCGCCCCCGAGGAGCGCGACTTCCTCGTCCGGGACATCGAGAGCCTAGATCTTGCTAGGATTGACCGGATCGTCCGA  207
UAP2  106 GAGGGCGCGTTCGCGCTCTGGGACGAGCTCTCGCCCGAGGACCGGGAGCTGCTCGTCAGGGACATCGAGAGCCTGGATCTTTCAAGGATTGATCGGATCATCCGG  210

UAP1  208 TGCTCGCTCAGATCACAAGGTGTTCCTTTGCCAGCCGTCGAGCCTGTGCCGGAGTCGAGTGTCTCGACCGTCGAAGATAGAACTCCTGAGGACAAGCAGAGGTGG  312
UAP2  211 CGCTCCCTTGGATCACAAGGATTCACTTTGCCCACCGTCGAGCCGGTGCCGGAGTCCAGCGTGTCCAAGGTGGAGGAGAGGTTGCCGGAGGACAAAGAACGGTGG  315

UAP1  313 TGGAAGAGGGGCTTGAAAGCCATTTCAGAGGGGAAATTGGCTGTTGTCCTTTTGGCTGGTGGTCAGGGAACAAGGCTTGGCAGTTCTGATCCTAAGGGATGCTTC  417
UAP2  316 TGGAAGAAGGGGCTGAAGGCCATTTCGGAGGGGAAGCTAGCCGTCGTCCTTTTAGCCGGTGGTCAGGGGACTCGGCTTGGTAGCTCTGATCCTAAAGGATGTTTC  420

UAP1  418 AGCATCGGGCTTCCATCTGGAAAGTCACTTTTCCAACTTCAAGCTGAACGAATTTTGTGCATACAGAAGCTGGCTGCTCAGTCCACTGATGGT---------ACT  513
UAP2  421 AGTATCGGACTTCCGTCTGGAAAGTCGCTTTTCCAACTCCAAGCTGAACGGATTTTGTGTGTTCAAAAGCTTGCTGCTCAATCAAGTGATAGTCCAAATAACACC  525

UAP1  514 CCACAAATACACTGGTATATAATGACTAGCCCCTTTACTGATGAAGCGACTCGAAAATTTTTGAAAGCCACAGATATTTTGGCTTAGAGCCTGACCAAGTAACA  618
UAP2  526 GTACCTATTCATTGGTATATAATGACCAGCCCCTTCACCGATGACATCACTCGCAAATTCTTCGAAAGCCGTAAATACTTTGGCTTAGAGGCAGACCAAGTGACA  630

UAP1  619 TTTTTCCAGCAAGGTACTATCCCATGTGTCTCAGCTGATGGAAGGTTTATTATGGAAACACCATACAAGGTAGCAAGGGCTCCTGATGGCAATGGTGGAGTGTAT  723
UAP2  631 TTTTTCCAACAAGGCACCCTTCCATGTGTTTCTGCTGATGGCAGATTTATTATGGAGACACCATACAAGGTAGCAAAGGCTCCTGATGGCAATGGTGGAGTTTAT  735

UAP1  724 GCTGCTCTAAAATCTCAAAGGTTGCTGGACGATATGGCTGGAAGAGGTGTGAAATATGTAGATTGCTATGGAGTTGACAATGTATTGGTCCGTGTTGCTGATCCA  828
UAP2  736 GCTGCCCTCAAGTCCAGAAGGTTGCTGGAAGATATGTCTTCACGGGGTGTAAAGTATGTAGATTGCTACGGGGTTGACAATGCACTGGTCCGCGTTGCTGATCCG  840

UAP1  829 ACATTCCTAGGATATTTCATTGACAAGGGCGTGTCTGCTGCTGCAAAGGTCGTAAGGAAGGCATATCCACAGGAGAAAGTTGGAGTGTTTGTTCAGCGTGGCAGG  933
UAP2  841 ACGTTCCTAGGTTACTTCATAGACAAAGCTGTATCTTCTGCTGCAAAGGTTGTTAGGAAGGCTTACCCACAAGAGAATGTTGGAGTATTTGTTCGACGAGGACGT  945

UAP1  934 GGTGGCCTCTTTCTGTAGTTGAGTACAGTGAAATGGATGCAGCTATGACTACTGAAATAAATCAAGGCACAGGGCGCCTTCGTTATTGTTGGAGCAATGTATGC 1038
UAP2  946 GGTGGACCTCTCTCTGTAGTTGAGTATAGTGAAATGGACGCAGCTATGGCTACTGAAATTAATCAGTCAACGGGACGCCTTCGTTATTGTTGGAGCAATATTTGC 1050

UAP1 1039 CTGCATATGTTTACTTTGGATTTTCTTAATCAAGTAACAAATAGTCTTGAAAAGGACAGCATTTACCATTTAGCAGAGAAGAAGATTCCTTCAATCCATGGGTAC 1143
UAP2 1051 TTGCATATGTTCACTTTGGATTTTTTGAATCAAGTAGCAAACAGCCTTGAGAAGGACAGTACTTATCATCTTGCTGAGAAGAAGATCCCTTCCATCCATGGGTAC 1155

UAP1 1144 ACGGCAGGCTTAAAGCTTGAACAGTTTATATTTGACGTGTTCACCTATTCTCCATCAACAGCTCTTTTTGAGATTTTGAGGGAGGAGGAATTTGCACCAGTAAAG 1248
UAP2 1156 GCAATGGGCTTGAAGCTTGAACAGTATATATTCGACGCGTTCAGTTACTCCCCATCCACAGCACTTTTTGAGGTTTTGCGGGAGGAGGAATTTGCGCCTGTAAAG 1260

UAP1 1249 AATGCTAATGGTGCAACTTATGATACTCCTGATAGTGCCAGATTAATGCTGCTCCGCCTTCACAGCCGATGGGTGGTAGCTGCAGGTGGCTTTTTGACTCATTCC 1353
UAP2 1261 AATGCGAATGGCGCGTCTTATGATACCCCTGATAGCGCAAAGCTGATGCTGCTTCGTCTCCATAGTAGATGGGTAGTTGCTGCTGGTGGCTTCTTGACCCATTCT 1365

UAP1 1354 GTGCCCTTGTATATGACAGGTGTTGAAGTTTCTCCACTTAGCTCTTATGCGGGAGAGAACCTGGAAGCCATATGCCGTGGACGGACATTCCATGCACCGAGTGAG 1458
UAP2 1366 GTCCCATTGTACATGACAGGTGTGGAAGTTTCTCCACTTAGCTCCTATGCTGGAGAAAACTTGGAAGCCATTTGCCGCGGACGGACATTCCATGCGCCGAGTGAG 1470

UAP1 1459 ATTTCATTTTAG 1470
UAP2 1471 ATTTCCTTCTAG 1482
```
