# Supplementary figures and images for: UDP-N-Acetylglucosamine Pyrophosphorylase 2 (UAP2) and 1 (UAP1) Perform Synergetic Functions for Leaf Survival in Rice
Source: Front Plant Sci. 2021 Jun 24;12:685102. doi: 10.3389/fpls.2021.685102 (PMC8264299; doi:10.3389/fpls.2021.685102)

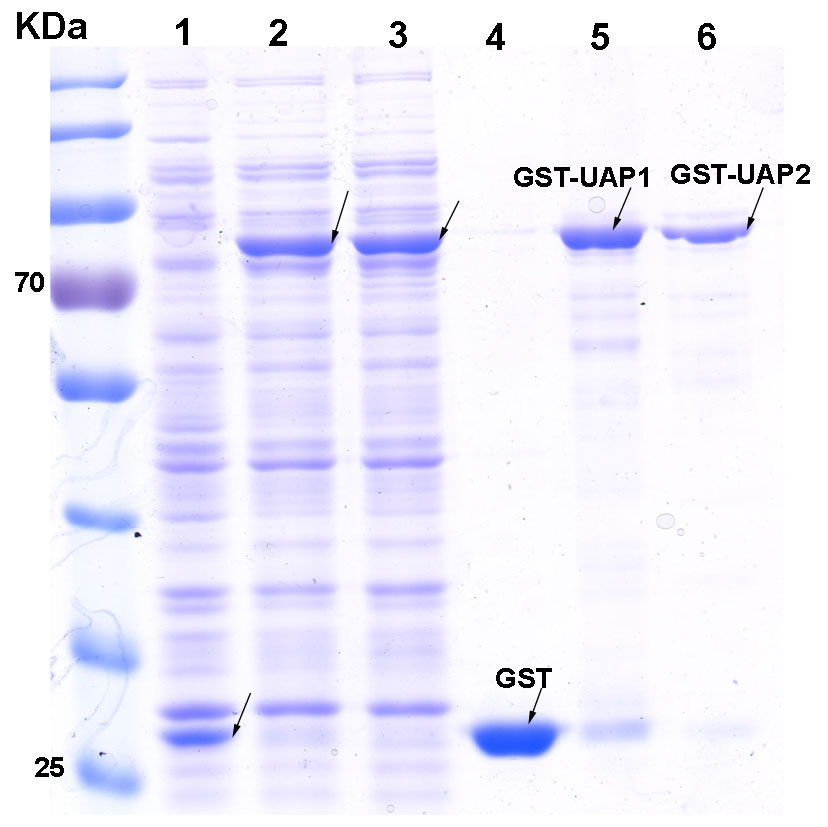

Supplement: Supplementary Figure 2 — SDS/PAGE of proteins. Lane 1, prestained protein ladder. Total soluble proteins from E.coli cells expressing control empty vector (lane 2), recombinant UAP1 (lane 3), and recombinant UAP2 (lane 4). Purified GST (lane 5), GST-UAP1 (lane 6), and GST-UAP2 (lane 7) proteins. Bands of GST, GST-UAP1, and GST-UAP2 are indicated by arrows. [file Image_2.JPEG]
