# Supplementary material for: UDP-N-Acetylglucosamine Pyrophosphorylase 2 (UAP2) and 1 (UAP1) Perform Synergetic Functions for Leaf Survival in Rice
Source: Front Plant Sci. 2021 Jun 24;12:685102. doi: 10.3389/fpls.2021.685102 (PMC8264299; doi:10.3389/fpls.2021.685102)
Supplement: Supplementary Table 1 — All primers used for qRT-PCR analysis. [file Data_Sheet_1.DOCX]

**Table S1** All primers used for qRT-PCR analysis

| Primer name | Forward primer | Reverse primer | Amplicon Length (bp) | Annealing (℃) | PCR efficiencey(%) | R^2^ |
| --- | --- | --- | --- | --- | --- | --- |
| *PR1a* | TGCATGTATGGACATGTAGTGTCATA | TACACTAAGCAAATACGGCTGACA | 133 | 60 | 88.6 | 0.998 |
| *PBZ1* | CGCAAGTCATGTCCTAAAGTCG | ATGCCATAGTAGCCATCCACG | 201 | 60 | 93.1 | 0.995 |
| *OsNAP* | GCAGGAGATGGCGAGGCAG | CCAGGTGCGCGAGCTGAAC | 122 | 60 | 104.5 | 0.991 |
| *Osl2* | GCAGACAACAAATCGCCAAAT | TCTCCAGCAACTCTAACCAGCAT | 107 | 60 | 97.7 | 0.998 |
| *Osh36* | ATCGCCAGAAGAAGACACCG | TTTCCCAACCAGCACACCC | 83 | 60 | 103.8 | 0.997 |
| *Osl43* | TGTGACAAGTGCTAATAATACATACGA | CCAGACCTTCCAAAGAATCCAAC | 108 | 60 | 93.0 | 0.999 |
| *Osl85* | TCCAGGATGTGATGAGGATTATTC | GCGTGCTGTAGTTCAGTCTGTAAAG | 121 | 60 | 96.7 | 0.998 |
| *ARF* | ATGAAAGGAAGACATGGCGG | TGGTGGTGGAACCTAAAGAGC | 126 | 60 | 95.2 | 0.999 |
| *UBC* | GTGCAGCGAGAAAAGTCAGC | GAACTTGCGGAGGAAGGAGAG | 172 | 60 | 95 | 0.998 |
| *Profilin-2* | CCAACTGGTCTTTTCCTTGGG | GGGGTCATCGGCTCATCATAG | 152 | 60 | 93.9 | 0.999 |
| *Actin1* | GGAAGTACAGTGTCTGGATTGGAG | TCTTGGCTTAGCATTCTTGGGT | 155 | 60 | 98.1 | 0.995 |

**Table S2** Primers for vector construction and confirmation of positive transgenic plants

| Primer name | Forward primer | Reverse primer |
| --- | --- | --- |
| GST-UAP1 | *cg****GGATCC***atggcggagatcgtggtggc | *cg****GAATTC***ctaaaatgaaatctcactcggtgc |
| GST-UAP2 | *cg****GGATCC***atgaaggagatagtggttgggtcg | *cg****GAATTC***ctagaaggaaatctcactcggcg |
| UAP1-YFP | ***cagtGGTCTCacaac***atggcggagatcgtggtggc | ***cagtGGTCTCataca***aaatgaaatctcactcggtgc |
| UAP2-YFP | ***cagtGGTCTCacaac***atgaaggagatagtggttgg | ***cagtGGTCTCataca***gaaggaaatctcactcggcg |
| *UAP2*-OE | ***cagtGGTCTCagttg***atgaaggagatagtggttgg | ***cagtGGTCTCaagag***ctagaaggaaatctcactcg |
| *Bar178* | AGAAACCCACGTCATGCCAGT | ACGCTCTACACCCACCTGCT |

Note: In primers ‘GST-UAP1’ and ‘GST-UAP2’, ‘***GGATCC***’ is the restriction enzyme sites BamHI, and ‘***GAATTC***’ is the the restriction enzyme sites EcoRI. In primers ‘UAP1-YFP’ and ‘UAP2-YFP’, ‘***GGTCTCacaac***’ and ‘***GGTCTCataca***’ are the Bsa I sites for YFP vector construction. In primer ‘*OsUAP2*-OE’, ‘***GGTCTCagttg***’ and ‘***GGTCTCaagag***’ are the Bsa I sites for over-expression vector construction.
